# Cardiovascular risk among 6-8-year-old children living in urban and rural communities in Ecuador: A cross-sectional analysis

**DOI:** 10.3389/fnut.2022.925873

**Published:** 2022-07-28

**Authors:** Signe Vargas-Rosvik, Nelly Lazo-Verdugo, Samuel Escandón, Cristina Ochoa-Avilés, Lucy Baldeón-Rojas, Angélica Ochoa-Avilés

**Affiliations:** ^1^Departamento de Biociencias, Universidad de Cuenca, Cuenca, Ecuador; ^2^Instituto de Investigación en Biomedicina, Universidad Central del Ecuador, Quito, Ecuador

**Keywords:** children, cardiovascular risk, dietary intake, anthropometry, Ecuador

## Abstract

Cardiovascular diseases have their origins in childhood. At least 20% of children and adolescents in Latin America are overweight or obese. However, little is known regarding the cardiovascular risk of young children living in the region. This paper aims to identify associations between socio-demographics, adiposity, and dietary intake with cardiometabolic risk among children between 6- and 8-years old living in urban and rural Andean regions of Ecuador. A cross-sectional study was conducted among 267 children attending elementary schools between February and August 2018. Sociodemographic data were collected using a structured interview. Bodyweight, height, and waist circumference were measured in duplicate; blood samples were taken after overnight fasting to determine blood lipids, hepatic enzymes, and adipokines; food intake data was assessed by two 24-h recalls administered to the guardians. Associations between cardiometabolic risk (i.e., blood lipids, hepatic enzymes, and adipokines) with sociodemographic characteristics, dietary intake, and waist circumference were tested using multiple hierarchical regression models. Twenty-nine percent of the children were overweight or obese, 12% had low HDL levels, and over 18% had high levels of LDL and triglycerides. Children living in the urban region had lower levels of HDL (β−4.07 mg/dL; 95% CI: −7.00; −1.15; *P* = 0.007) but higher levels of LDL cholesterol (β 8.52 mg/dL; 95% CI: 1.38; 15.66; *P* = 0.019). Hepatic enzymes were also higher among urban children (SGOT: β% 22.13; 95% CI: 17.33; 26.93; *P* < 0.001; SGPT: β 0.84 U/L; 95% CI: 0.09; 1.59; *P* = 0.028). Leptin blood levels were higher (β% 29.27; 95% CI: 3.57; 54.97; *P* = 0.026), meanwhile adiponectin plasma concentrations were lower among urban children (β%−103.24; 95% CI: −58.9; −147.58; *P* = < 0.001). Fiber intake was inversely associated with total cholesterol (β−9.27 mg/dL; 95% CI –18.09; −0.45; *P* = 0.040) and LDL cholesterol blood levels (β−9.99 mg/dL; 95% CI: −18.22; −1.75; *P* = 0.018). Our findings demonstrate that young children are at high cardiovascular risk; if no actions are taken, the burden of non-communicable diseases will be substantial. The differences in risk between rural and urban areas are evident; urbanization might predispose children to a different reality and, in most cases, result in poor habits.

## Introduction

Cardiovascular diseases (CVD) are the leading causes of death worldwide, responsible for around 31% of global deaths. Although CVDs have their onset during adulthood, their pathogenic process begins during childhood and adolescence, a critical period for detecting CVD risk factors such as obesity, dyslipidemia, high blood pressure, metabolic syndrome, non-alcoholic fatty liver disease, atherogenic diet, and physical inactivity (1, 2).

Evidence demonstrates that the burden of CVD risk factors among children and adolescents is high. In Latin America, over 20% of children and adolescents are overweight or obese, a situation which often coexists with other cardiovascular risk factors (3, 4). The American Heart Association data shows that the fraction of children and adolescents in the United States with atherogenic dyslipidemia has increased in the last decade. About 25% of adolescents have high triglycerides and low high-density lipoprotein (HDL) blood levels (5), and dyslipidemia is seven times higher among obese children than their lean counterparts (6). Several factors play a role in dyslipidemia development among obese children; insulin resistance and visceral adiposity are the most important factors (5). Pediatric non-alcoholic fatty liver disease is the most common chronic liver disorder in children and adolescents with obesity (5, 7). Worryingly, children with non-alcoholic fatty liver disease often have dyslipidemia, and the severity of the disease is linked to an increased prevalence of CVD risk in children (7).

Molecular factors produced by adipose tissue play a critical role in cardiovascular risk among obese populations. Obesity is a chronic state of inflammation related to adipokines imbalance (i.e., high blood levels of leptin and low levels of adiponectin), which is a critical factor for cardiovascular disease (8–10). Leptin prevents lipid accumulation in peripheral tissues and stimulates the metabolism of fatty acids and glucose in skeletal muscle (11, 12). However, leptin’s stimulatory effect on fatty acid oxidation is absent in obese people, and leptin resistance is one of the causal factors of cardiovascular complications in obesity (11, 12). Adiponectin regulates energy homeostasis, glucose, and lipid metabolism. Blood levels of adiponectin are decreased in patients with obesity, diabetes, and coronary heart disease (13, 14).

Inadequate dietary intake, physical inactivity, socioeconomic factors, and urbanization are important risk factors for dyslipidemia and cardiovascular diseases (15–17). In Latin America, the consumption of energy-dense, nutrition-poor foods has dramatically increased, while the intake of fruit, vegetables, whole grains, cereals, and legumes has decreased (3, 4). With food globalization, the correlation between income and dietary patterns has changed dramatically; lower-income countries consume higher percentages of calories from fat and sugars; this could predispose the population to a double burden of malnutrition, where undernutrition coexists with overweight and obesity (18). Chile, Mexico, and Argentina are the countries with faster development, the sharpest rise in processed foods and processed beverage sales and the highest prevalence of cardiovascular risk factors (3, 4).

Regarding cardiovascular risk status and its behavioral and social determinants, little is known among young children in Latin America. According to a systematic review, the available research has quantified overweight and obesity prevalence in the region (19). However, information regarding cardiovascular risk factors, such as abnormal blood lipids, hepatic profile, and adipokines, is scarce among young children. Furthermore, associations between the level of urbanization, sociodemographic factors, and eating behavior with cardiometabolic risk are poorly understood among young Latin American children (8, 20). Understanding the cardiovascular risk status and its influential factors in this specific population is essential to providing informed recommendations. Thus, this paper aims to: (i) determine weight status and abdominal adiposity; (ii) estimate cardiometabolic risk by measuring blood lipid profile, adipokines, serum glutamic oxaloacetic transaminase (SGOT), and serum glutamic pyruvic transaminase levels (SGPT); (iii) characterize dietary intake; and (iv) identify associations between socio-demographics, adiposity, and dietary intake with cardiometabolic risk among 6–8-year old children living in urban and rural areas in Ecuador’s Andean region.

This paper is the first report of the project “The Andean Microbiome,” which aims to determine the factors associated with microbiota patterns of children 6–7 years of age living in urban and rural areas of the Andean highlands in Ecuador.

## Materials and methods

### Study design, setting and sampling

A cross-sectional study was conducted between February and August 2018 with a convenient sample of 267 children aged 6–8 years living in the Andean region of Ecuador. The participants were recruited at three schools located at altitudes over 2.100 m.a.s.l; one was in the urban area, and two were in the rural area. The urban school was located in Cuenca, a city in the country’s southern Andean region, where 20% of the population is classified as poor based on unsatisfied basic needs, and 2.5% of the inhabitants are illiterate (21). The rural schools were located in the northern Andean region (Quito) in parishes with poverty levels ranging between 40 and 57% and illiteracy rates of 5% (22–25). In each school, all the 6–8-year-old children were invited to participate; only children with signed informed consent by their parents/guardians were included. This study was approved by the Ethics Committee of Universidad San Francisco de Quito code: 2017-152-M.

### Measurements

Sociodemographic data, blood sampling, and weight status measurements were taken at school during regular school hours with the parent/guardian present. Dietary intake data were collected at the participants’ homes on dates arranged beforehand with the guardian. Dietary intake data could not be collected when the guardian/tutor was unavailable. Data were collected by trained staff under standardized procedures.

### Sociodemographic data

Sociodemographic characteristics were collected using a structured interview applied to the guardian. The interview included questions from the National Institute of Statistics and Census, such as age, home address, time living in the current residence, child ethnicity and the guardian’s education level (26).

### Anthropometry

A trained researcher measured anthropometric variables in duplicate, considering a third measurement only if the difference between the first two was greater than 0.5 kg for weight and 0.5 cm for height, according to standardized procedures based on the INBIOMED Manual (27). During the measurements, children wore light clothes and no shoes. Weight was measured to the nearest Kg using a mechanical calibrated scale (SECA 760), and height was measured to the nearest mm, using a calibrated stadiometer SECA 213 (Seca, Hamburg, Germany). Waist circumference was measured to the nearest mm using a tape measure [SECA (28)]. Body mass index for-age Z-score was calculated was calculated using the World Health Organization’s (WHO) macros for Stata [WHO (29)]. Subsequently, according to WHO cutoff points, children were classified as underweight, normal weight, overweight, and obese (29).

### Cardiometabolic risk profile

Blood samples were taken in the morning after overnight fasting by venipuncture at the antecubital vein using anticoagulant-free tubes. All the samples were centrifuged at 3,400 rpm for 8 min to obtain blood serums and were transported on dry ice to the laboratory, where they were stored at −80°C until processing, which took no more than 15 days. Total cholesterol, triglycerides, SGOT, and SGPT, were measured using commercial kits (Human, Wiesbaden, Germany) in a spectrophotometer Eppendorf PCP6121 (Eppendorf, Hamburg, Germany). HDL cholesterol was quantified by precipitation of lipoproteins (Human, Wiesbaden, Germany). The Friedewald formula was used to calculate LDL-cholesterol (plasma LDL concentrations = plasma total cholesterol concentrations – plasma HDL concentrations – plasma TAG concentrations/5). Leptin and adiponectin were determined by the ELISA sandwich technique using DIAsource kits (DIAsource ImmunoAssays S.A., Belgium); readings were performed in a H.S. Human ELISA’ lector equipment (Human, Wiesbaden, Germany). Blood lipid values were categorized according to reference values for children (30). SGPT and SGOT were classified in acceptable and high ranges according to Human SGPT and SGOT kits. Values < 37 U/L for men and < 31 U/L for women for SGOT, and < 42 U/L for men and < 32 U/L for women SGPT were classified as normal.

### Dietary intake

Food intake data were assessed by two 24-h recalls administered using the multi-pass method (31, 32); different interviewers, randomly assigned by a principal investigator, applied the recalls on a weekday and a weekend day. Portion sizes were estimated by employing local standardized utensils (33).

A list of all the recipes reported in the 24-h recalls was constructed, and food ingredients and quantities were estimated using standardized recipes from previous studies (33, 34). In the case of unavailable recipes, these were prepared in duplicate following the details provided by the participant. The ingredients and total recipe weight were measured to obtain an average for each recipe.

In Ecuador, an up-to-date food composition database does not exist. For this reason, nutrient intake was calculated using a compiled food composition database (33). The database was constructed by searching the United States [USDA (35)], Mexican [INNSZ (36)], Central American [INCAP/OPS (37)], and Peruvian [CENAN/INS (38)] databases. Nutrient content of the processed and pre-packaged food was obtained from nutrition facts labels and was included in the compiled food composition database.

The data from the 24-h recalls were entered using *MikunaSoft*, a software developed by this paper’s authors. The standardized recipes and the compiled food composition database were uploaded to the software to estimate the participants’ dietary intake. Total energy intake (in kilocalories/day), carbohydrates, total fat, protein, and fiber intake (in grams/day) were estimated using the average of the two 24-h recalls. Macronutrients and fiber intake are reported as the percentage of energy intake per day (E%/day). In addition, nutrient intake was categorized according to the dietary reference intake cutoffs as insufficient, adequate, and high. Dietary reference intake for 4 to 8-year-old children is 45–65 E%/d for carbohydrates, 10–30 E%/d for protein, and 25–35 E%/d for total fat; finally, the adequate intake for fiber is 25 g/d (39).

All the recipes and food items reported in the 24-h recalls were categorized into four groups according to the NOVA food framework: (i) unprocessed or minimally processed foods, (ii) culinary ingredients, (iii) processed foods, and (iv) ultra-processed foods (40). The E%/day was calculated from each NOVA food group.

### Data analysis

Data were analyzed using Stata (Stata Statistical Software: Release 13. College Station, TX, United States: Stata Corp LLC), and figures were obtained using RStudio [RStudio Team (41). RStudio: Integrated Development for R. RStudio, PBC, Boston, MA, United States].^1^ A significance level of 5% was considered for the statistical tests. Continuous variables were reported as mean with standard deviation, while categorical variables were displayed in frequency tables and bar charts. The distribution of continuous variables (and their possible transformations) was assessed using the ladder and gladder commands in Stata.

Differences in continuous variables between urban and rural areas for descriptive tables were tested using the Student’s *T*-Test (with unequal variances when appropriated) or Wilcoxon Rank Sum test depending on the variables’ distribution. Chi-square tests were applied to compare frequency distributions of categorical variables between urban/rural areas.

Multiple hierarchical regression models adjusted for age and sex with three steps were applied to identify predictors significantly associated with cardiometabolic risk outcomes (i.e., blood lipids, hepatic enzymes, and adipokines). Step 1 included residence (urban/rural) and de education level of the guardian as independent variables. In Step 2, BMI for age z-score and waist circumference were introduced; finally, macronutrients and fiber intake were included as independent variables in Step 3. Firstly, bivariate models were built with each cardiometabolic risk outcome as a dependent variable and sociodemographics, BMI for age z-score, waist circumference and nutrients as independent variables. Only independent variables significantly associated (*P* < 0.10) with the outcomes in bivariate models were included in the hierarchical adjusted model. For each model, regression diagnoses were evaluated (i.e., residuals versus fitted values plots); when unequal error variances or outliers were detected, the dependent variables were log-transformed, and the corresponding Beta coefficients were back-transformed and expressed as percentage differences. Multicollinearity between the independent variables was analyzed using the variance inflation factor (VIF), which showed a small risk of multicollinearity. The final models were tested for heterogeneity and properly corrected using robust standard deviation.

## Results

A total of 267 children were recruited; the participants’ average age was 6.7 ± 0.7 years, 36.3% lived in the urban area, and 47% were female (Table 1). Most guardians identified their children as mestizos (92%); 53% of the guardians completed secondary education, while 29% completed primary education only. There were no differences in age, sex or parental level of education between urban and rural children (Table 1).

**TABLE 1:** Sociodemographic characteristics of the study participants.

	Total *n* = 267		Urban *n* = 97		Rural *n* = 170		
			
	*n*	%	*N*	%	*n*	%	P*
Sex							0.336
Female	126	47.2	42	43.3	84	49.4	
Male	141	52.8	55	56.7	86	50.6	
Ethnicity *n* = 259							0.011
Mestizo (European and Indigenous ancestry)	238	91.9	81	86.2	157	95.2	
Other^a^	21	8.1	13	13.8	8	4.8	
Educational level of the guardian *n* = 260							0.109
None/Primary education	81	31.1	25	26.0	56	34.2	
Secondary education	138	53.1	51	53.1	87	53.1	
Higher education/Master’s degree	41	15.8	20	20.8	21	12.8	

* Differences were tested using the Pearson’s Chi-square non-parametric test.

^a^ Other ethnic groups: white, Afro-descendant and Indigenous.

### Anthropometry

**FIGURE 1:**
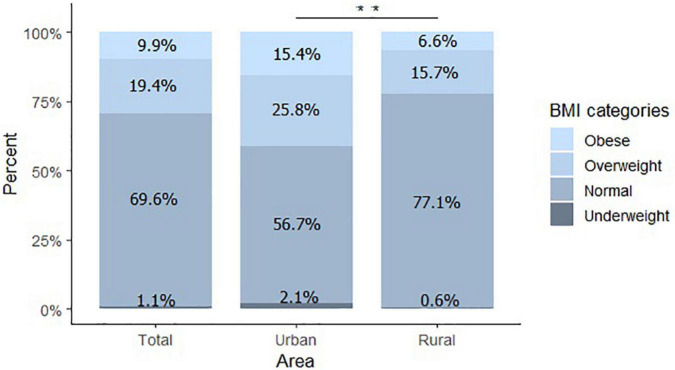
Body mass index classification. Percentage of children obese, overweight, normal, and underweight. Total *n* = 263, Urban *n* = 97, Rural *n* = 166. ^**^P < 0.01.

The mean BMI Z-score was 0.5 ± 1.1, 19% of the children were overweight, and 10% were obese. Bodyweight, height, waist circumference, and BMI Z-score were significantly higher among children living in the urban area (*P* < 0.01) (Table 2). Likewise, overweight and obesity prevalence was significantly higher among children in the urban area than those in the rural area (26 vs. 16% and 16 vs. 7%, respectively; *P* = 0.005) (Figure 1).

**TABLE 2:** Blood lipid, hepatic profile and weight status of the study participants.

	Total *n* = 264		Urban *n* = 97		Rural *n* = 167		
			
	*Mean*	*SD*	*Mean*	*SD*	*Mean*	*SD*	*P*
Body weight (Kg) (*n* = 267)	23.8	4.7	25.3 (*n* = 97)	5.2	23.0 (*n* = 170)	4.1	<0.001^a^
Body height (cm) (*n* = 267)	118.9	5.5	120.6	5.7	118.0	5.1	<0.001^a^
BMI for age z-score (*n* = 263)	0.5	1.1	0.8	1.3	0.4 (*n* = 166)	1.0	0.008^b^
Waist circumference (cm)	58.67	6.69	60.8	7.44	57.46	5.92	<0.001^a^
Triglycerides (mg/dL)	86.8	42.8	92.0	47.9	83.8	39.3	0.254^a^
Total Cholesterol (mg/dL)	175.3	29.1	180.2	30.1	172.5	28.3	0.041^a^
HDL Cholesterol (mg/dL)	53.0	11.4	50.2	11.3	54.6	11.2	0.003^a^
LDL Cholesterol (mg/dL)	105.0	27.1	111.5	27.9	101.2	26.0	0.003^a^
SGOT (U/L)	23.9	5.0	27.3	5.0	21.9	3.8	<0.001^b^
SGPT (U/L)	10.4	3.0	11.3	3.7	9.8	2.3	0.001^a^
Leptin (ng/ml) (*n* = 129)	1.7	2.4	2.6 (*n* = 40)	3.5	1.4 (*n* = 89)	1.7	0.005^b^
Adiponectin (μg/ml) (*n* = 129)	7.4	6.9	3.8 (*n* = 40)	5.8	9.1 (*n* = 89)	6.7	<0.001*^b^*

SD, standard deviation; HDL, high density lipid; LDL, low density lipid; SGOT, serum glutamic oxaloacetic transaminase; SGPT, serum glutamic pyruvic transaminase; BMI, body mass index.

^a^ Differences were estimated using the parametric student’s test.

^b^ Differences were estimated using the Wilcoxon Rank-sum non-parametric test.

### Cardiometabolic risk profile

The mean values of blood lipids, SGOT, SGPT and adipokines are shown in Table 2. Mean triglycerides (86.8 ± 42.8 mg/dL) and total cholesterol blood levels (175.3 ± 29.1 mg/dL) were above the adequate reference values. Blood levels of total cholesterol, LDL cholesterol, SGOT, SGPT, and leptin were significantly higher among children living in the urban area (*P* < 0.01). On the other hand, the mean HDL cholesterol and adiponectin blood concentrations were lower among urban children (*P* < 0.01).

**FIGURE 2:**
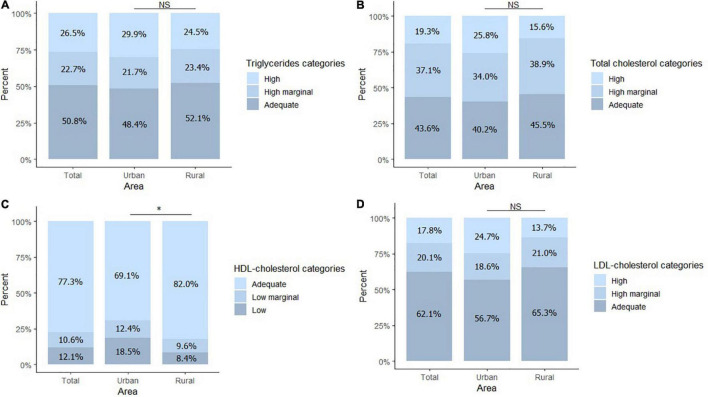
Blood lipid profile. **(A)** Percentage of children within high, marginal high, and adequate values for triglycerides. **(B)** Percentage of children within high, marginal high, and adequate values for total cholesterol. **(C)** Percentage of children within low, marginal low, and adequate values for HDL-cholesterol. **(D)** Percentage of children within high, marginal high, and adequate values for LDL- cholesterol. Total *n* = 264, Urban *n* = 97, Rural *n* = 167. *P < 0.05. NS, no significant differences.

A substantial proportion of the children showed marginally high or high levels of total cholesterol (56%), triglycerides (49%), and LDL cholesterol (38%). Nearly a quarter of the participants (23%) had marginally low or low levels of HDL cholesterol (Figure 2). SGPT values were between the normal ranges for all children, and 4.2% of the participants showed high values of SGOT. A higher proportion of children in the urban area had low levels of HDL cholesterol in comparison with children from the rural area (18.5 vs. 8.4%, *P* = 0.003).

### Dietary intake

Dietary intake data were available for 145 children (*n* = 90 from the urban area and *n* = 55 from the rural area). Total energy intake (total sample mean 10757.1 ± 461.4 Kcal) in the urban area was significantly higher than in the rural area (1961.5 kcal/day vs. 1422.6 kcal/day; *P* < 0.001).

**FIGURE 3:**
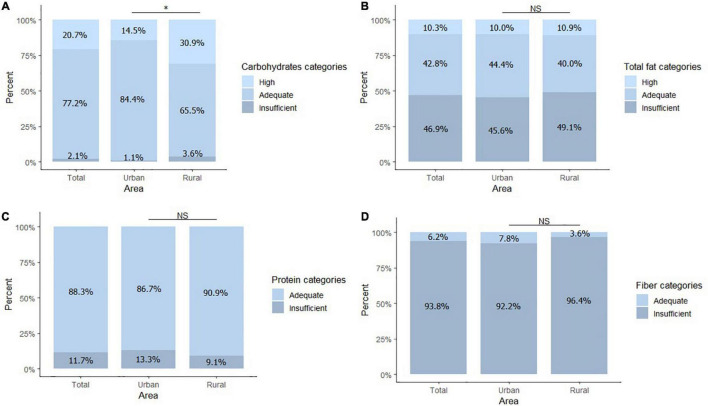
Dietary intake according to recommendation. **(A)** Percentage of children within high, adequate, and insufficient carbohydrate consumption category. **(B)** Percentage of children within the high, adequate, and insufficient total fat consumption category. **(C)** Percentage of children within the adequate and insufficient protein consumption category. **(D)** Percentage of children within the adequate and insufficient fiber consumption category. Total *n* = 145, Urban *n* = 90, Rural *n* = 55. The difference in sample size among the study variables is explained by the fact that not all parents in the rural area were able to schedule a meeting for the 24-recall. *P < 0.05. NS, no significant differences.

Carbohydrate intake provided, on average, 60% of the daily energy intake, followed by fat (26%) and proteins (12.5%). The percentage of carbohydrate consumption doubled in the rural area compared to the urban area (31 vs. 14% *P* = 0.028) (Table 3). Total fat intake was within the recommended range for 43% of the children, and 47% percent had an insufficient fat intake. Most of the children had an adequate protein intake (88%). Ninety-four percent of the participants had insufficient fiber intake, with no significant difference between areas (Figure 3).

**TABLE 3:** Dietary intake of the study participants.

	Total *n* = 145		Urban *n* = 90		Rural *n* = 55		
			
Dietary intake	*Mean*	*SD*	*Mean*	*SD*	*Mean*	*SD*	*P*
Total energy (kcal/day)	1757.1	461.4	1961.5	378.9	1422.6	384.0	<0.00^a^
Total fat intake (%E/day)	26.1	6.4	26.6	5.9	25.2	7.2	0.229^a^
Total protein (%E/day)	12.5	2.4	12.4	2.3	12.7	2.5	0.622^b^
Total carbohydrate (%E/day)	59.8	7.0	59.5	6.3	60.3	8.2	0.542^a^
Total fiber (%E/day)	1.6	0.6	1.5	0.5	1.8	0.7	0.037^a^
**NOVA^c^**							
Minimally processed food (%E/day)	49.6	11.7	48.2	10.6	51.8	13.2	0.071^a^
Culinary ingredients (%E/day)	14.6	6.7	14.9	6.8	14.0	6.5	0.306^b^
Processed food (%E/day)	15.3	7.3	15.0	7.3	15.6	7.3	0.646^a^
Ultra-processed food (%E/day)	20.6	11.9	21.0	12.2	20.1	11.5	0.675^a^

SD, standard deviation; %E/day, energy percentage per day.

^a^ Differences were estimated using the parametric student’s test.

^b^ Differences were estimated using the Wilcoxon Rank-sum non-parametric test.

^c^ NOVA: NOVA food framework: (i) unprocessed or minimally processed foods, (ii) culinary ingredients, (iii) processed foods, and (iv) ultra-processed foods (40). The E%/day was calculated from each NOVA food group. The difference in sample size among the study variables is explained by the fact that not all parents in the rural area were able to schedule a meeting for the 24-recall.

Fifty percent of daily energy was obtained from minimally processed or unprocessed food/ingredients, followed by ultra-processed foods (21% E/day). Culinary ingredients and processed foods contributed 14 and 15% E/day, respectively. There was no significant difference in NOVA groups intake between urban and rural areas (Table 3).

### Associations between blood lipid, hepatic and adipokine profile and sociodemographic characteristics, BMI z-score, and food intake variables

Table 4 displays the results of the hierarchical models. Sex was associated with cardiometabolic risk after adjustment for anthropometrics. Female children had higher levels of triglycerides (β 16.13 mg/dL; 95% CI: 5.96; 26.30; *P* = 0.002); and leptin serum concentration was 62% higher among female children in comparison with males (β% 62.32; 95% CI: 39.19; 85.44; *P* < 0.001). Living in the urban area was associated with a higher cardiometabolic risk (after adjusting for anthropometric variables; Step 2). Children living in the urban region had lower blood levels of HDL cholesterol (β−4.07 mg/dL; 95% CI: −7.00; −1.15; *P* = 0.007) but higher levels of LDL cholesterol (β 8.52 mg/dL; 95% CI: 1.38; 15.66; *P* = 0.019). Hepatic enzymes were also higher among urban children in comparison with their rural counterparts (SGOT β% 22.13; 93% CI: 17.33; 26.95; *P* < 0.001. SGPT β 0.84 U/L; 95% CI: 0.09; 1.59; *P* = 0.028). Leptin blood levels were 29% higher (β% 29.27; 95% CI: 3.57; 54.79; *P* = 0.026), meanwhile adiponectin plasma concentrations were 103% lower among urban children (β%−103.24; 95% CI: −147.58; −58.9; *P* = < 0.001). Abdominal adiposity measured by waist circumference was positively associated with blood triglycerides (β 2.68 mg/dL; 95% IC 0.74; 4.61; *P* = 0.007), SGPT (β 0.17 U/L; 95% CI: 0.05; 0.28; *P* = 0.004), and leptin plasma concentrations (β% 7.05; 95% CI: 2.52; 11.58; *P* = 0.003).

**TABLE 4:** Associations between blood lipids, hepatic enzymes and adipokines with sociodemographic, anthropometrics, and nutrient intake data*.

*Step*	*Predictors*	Triglycerides^b^			Total cholesterol			LDL cholesterol			HDL cholesterol^b^		
					
		β	95*% CI*	*P*	β	95*% CI*	*P*	β	95*% CI*	*P*	β	95*% CI*	*P*
**1**	Age (years)	2.87	−3.70; 9.44	0.390	4.05	−1.09; 9.20	0.122	1.31	−3.45; 6.07	0.589	1.93	−0.06; 3.93	0.058
	Sex (0 male, 1 female)	**15**.**55**	**4**.**93; 26**.**17**	**0**.**004**	4.82	−2.27; 11.92	0.182	1.86	−4.72; 8.44	0.579	−1.73	−4.49; 1.03	0.218
	Residence (0 rural, 1 urban)	**–**	**–**	**–**	5.08	−2.42; 12.57	0.184	**9**.**90**	**2**.**95; 16**.**85**	**0**.**005**	−**5**.**27**	−**8**.**18;**−**2**.**35**	**<0**.**001**
	Educational level of the guardian^a^	–	–	–	4.49	−0.90; 9.87	0.102	–	–	–	–	–	–
**2**	Age (years)	−2.24	−9.49; 5.00	0.543	4.45	−1.16; 10.05	0.120	1.72	−3.48; 6.92	0.516	**2**.**78**	**0**.**65; 4**.**91**	**0**.**011**
	Sex (0 male, 1 female)	**16**.**13**	**5**.**96; 26**.**3**	**0**.**002**	4.28	−2.81; 11.38	0.236	1.43	−5.16; 8.03	0.669	−1.85	−4.55; 0.86	0.180
	Residence (0 rural, 1 urban)	–	–	–	3.48	−4.19; 11.15	0.372	**8**.**52**	**1**.**38; 15**.**66**	**0**.**019**	−**4**.**07**	−**7**.**00;**−**1**.**15**	**0**.**007**
	Educational level of the guardian^a^	**–**	**–**	**–**	4.08	−1.29; 9.45	0.136	**–**	**–**	**–**	–	–	–
	BMI Z-score	−0.90	−10.78; 8.98	0.857	1.70	−5.67; 9.06	0.651	1.60	−5.32; 8.51	0.650	0.14	−2.69; 2.98	0.921
	Waist circumference (cm)	**2**.**68**	**0**.**74; 4**.**61**	**0**.**007**	0.12	−1.15; 1.39	0.855	0.07	−1.12; 1.27	0.904	−0.46	−0.95; 0.03	0.064
**3**	Age (years)	**–**	**–**	**–**	2.86	−5.02; 10.75	0.474	0.62	−6.67; 7.91	0.866	**–**	**–**	**–**
	Sex (0 male, 1 female)	**–**	**–**	**–**	9.06	−0.76; 18.89	0.070	5.96	−3.21; 15.13	0.201	**–**	**–**	**–**
	Residence (0 rural, 1 urban)	**–**	**–**	**–**	−0.26	−10.94; 10.42	0.961	4.72	−5.14; 14.58	0.345	**–**	**–**	**–**
	Educational level of the guardian^a^	**–**	**–**	**–**	5.44	−1.89; 12.78	0.144	**–**	**–**	**–**	**–**	**–**	**–**
	BMI Z-score	**–**	**–**	**–**	2.02	−8.43; 12.48	0.702	2.72	−7.03; 12.48	0.582	**–**	**–**	**–**
	Waist circumference (cm)	**–**	**–**	**–**	0.18	−1.57; 1.92	0.841	0.13	−1.50; 1.76	0.874	**–**	**–**	**–**
	Fiber (%E/day)	–	–	–	−**9**.**27**	−**18**.**09;**−**0**.**45**	**0**.**040**	−**9**.**99**	−**18**.**22;**−**1**.**75**	**0**.**018**	–	–	–

***Step***	***Predictors***	**SGOT** *^b,c^*			**SGPT** ^b^			**Leptin** *^b,c^*			**Adiponectin** *^b,c^*		
					
		***B %***	**95*% CI***	***P***	**β**	**95*% CI***	***P***	***B %***	**95*% CI***	***P***	***B %***	**95*% CI***	***P***

**1**	Age (years)	−0.74	−3.93; 2.45	0.648	0.44	−0.04; 0.92	0.070	4.13	−17.36; 25.62	0.705	14.12	−15.76; 44.00	0.351
	Sex (0 male, 1 female)	−0.65	−5.05; 3.75	0.773	0.19	−0.54; 0.92	0.607	**66**.**25**	**37**.**55; 94**.**95**	**<0**.**001**	−0.77	−40.39; 38.85	0.969
	Residence (0 rural, 1 urban)	**22**.**29**	**17**.**64; 26**.**94**	**<0**.**001**	**1**.**36**	**0**.**50; 2**.**23**	**0**.**002**	**49**.**42**	**18**.**02; 80**.**82**	**0**.**002**	−**110**.**11**	−**153**.**58;**−**66**.**65**	**<0**.**001**
	Educational level of the guardian^a^	3.01	−0.33; 6.35	0.077	–	–	–	–	–	–	–	–	–
**2**	Age (years)	−0.68	−4.19; 2.84	0.705	0.14	−0.37; 0.65	0.586	−5.98	−24.89; 12.93	0.533	23.18	−9.72; 56.08	0.166
	Sex (0 male, 1 female)	−0.64	−5.08; 3.80	0.777	0.22	−0.45; 0.90	0.517	**62**.**32**	**39**.**19; 85**.**44**	**<0**.**001**	−0.05	−39.75; 39.66	0.998
	Residence (0 rural, 1 urban)	**22**.**13**	**17**.**33; 26**.**93**	**<0**.**001**	**0**.**84**	**0**.**09; 1**.**59**	**0**.**028**	**29**.**27**	**3**.**57; 54**.**97**	**0**.**026**	−**103**.**24**	−**147**.**58;**−**58**.**9**	**<0**.**001**
	Educational level of the guardian^a^	3.03	−0.33; 6.40	0.077	**–**	**–**	**–**	**–**	**–**	**–**	**–**	**–**	**–**
	BMI Z-score	0.92	−3.70; 5.53	0.695	0.16	−0.44; 0.77	0.594	7.66	−17.71; 33.03	0.551	18.85	−24.64; 62.35	0.393
	Waist circumference (cm)	−0.07	−0.87; 0.72	0.858	**0**.**17**	**0**.**05; 0**.**28**	**0**.**004**	**7**.**05**	**2**.**52; 11**.**58**	**0**.**003**	−5.82	−13.63; 2.00	0.143
**3**	Age (years)	**–**	**–**	**–**	**–**	**–**	**–**	**–**	**–**	**–**	**–**	**–**	**–**
	Sex (0 male, 1 female)	**–**	**–**	**–**	**–**	**–**	**–**	**–**	**–**	**–**	**–**	**–**	**–**
	Residence (0 rural, 1 urban)	**–**	**–**	**–**	**–**	**–**	**–**	**–**	**–**	**–**	**–**	**–**	**–**
	Educational level of the guardian^a^	**–**	**–**	**–**	**–**	**–**	**–**	**–**	**–**	**–**	**–**	**–**	**–**
	BMI Z-score	**–**	**–**	**–**	**–**	**–**	**–**	**–**	**–**	**–**	**–**	**–**	**–**
	Waist circumference (cm)	–	–	–	–	–	–	–	–	–	–	–	–
	Fiber (%E/day)	–	–	–	–	–	–	–	–	–	–	–	–

*Analysis performed using hierarchical regression models with blood lipids, hepatic enzymes and adipokines as dependent variables; Age, Sex, Place of residence and Education level of the guardian as independent variables in the first step; BMI Z-score and waist circumference in step 2; and nutrients in Step 3. Only variables significantly associated with the dependent variables in bivariate linear regression models were included in the hierarchical regressions.

^a^ Education level of the guardian: 0 = None/primary education, 1 = Secondary education, 2 = Higher education/Master’s degree.

^b^ Step 3 is not shown as no significant association was found in the bivariate analysis with any nutrient.

^c^ Dependent variables log-transformed, the results are presented as B% to enhance interpretability. –Variables not significantly associated with the dependent variables in bivariate linear regression models.

**Bold:** Significant associations in the hierarchical models.

Fiber was the only nutrient associated with cardiometabolic risk after adjustment for sociodemographic and anthropometric variables (Step 3). Every additional percentage of daily energy intake obtained from fiber was associated with a lower concentration of total cholesterol (β−9.27 mg/dL; 95% CI –18.09; −0.45; *P* = 0.040) and LDL blood levels (β 9.99 mg/dL; 95% CI: −18.22, −1.75; *P* = 0.018).

## Discussion

This study found that young Ecuadorian children living in urban and rural areas are at high cardiovascular risk. Three out of ten children were overweight or obese. A quarter of the participants had low HDL values, and an important proportion of the children showed high triglycerides, total cholesterol, and LDL blood levels. Regarding dietary intake, carbohydrates were the primary energy source, and 20% of participants with available data consumed an excess of carbohydrates. Worryingly, refined carbohydrates seem to be an essential constituent of young children’s diet. The energy obtained from dietary fiber did not even reach 2% per day. Children living in urban areas appear to be at higher cardiovascular risk. Overweight and obesity were twice as prevalent among urban children; unfavorable blood lipid profiles were detected more frequently in children from urban areas; hepatic enzymes and leptin blood levels were higher, and adiponectin was lower among urban children when compared with their rural counterparts. Furthermore, living in urban areas was associated with having higher values of LDL cholesterol, SGOT, SGPT, and adiponectin blood levels but lower HDL cholesterol levels. Finally, visceral fat seems to be a critical risk factor associated with high triglycerides SGPT and leptin blood levels among young children.

### Anthropometry

Our overweight/obesity estimate (29%) is in line with the one reported in the Ecuadorian health survey conducted in 2012 (29.9%) (21) but lower than the estimate of the national survey conducted in 2018 (35.4%) (42). In any case, the overweight/obesity burden in the studied area lies within the Latin American prevalence rate (18.9 to 36.9%) among school-age children (19). It is a matter of concern since childhood overweight and obesity are associated with an increased risk of cardiovascular diseases, type 2 diabetes, and high blood pressure (43, 44).

### Blood lipids profile

Our study’s faction of children with low HDL cholesterol levels is in line with reports among children in the United States (12 vs. 12.8%) (45). However, the prevalence of elevated levels of total cholesterol, triglycerides, and LDL cholesterol is higher in our study population than in reports from the United States (45). Unfortunately, we did not find relevant studies in Latin America on the general pediatric population. Our data highlights the need to conduct population-based studies in the region on larger samples to quantify the risk; the evidence suggests that 40 to 50% of children with elevated blood lipid levels would continue this trend in adulthood and, consequently, have a higher cardiovascular risk (46). A possible explanation for the alterations in the lipid profile could be an unbalanced diet. We identified an inverse correlation between fiber intake and total and LDL cholesterol levels, a result in line with a systematic review that reported that the consumption of whole grains (a source of fiber) has the potential to lower LDL and total cholesterol (47). Our data and reports on Ecuadorian children and adolescents have shown that Ecuadorians’ diet is poor in whole grains and fiber (21, 33). Adequate fiber intake has several health benefits, especially when it starts during childhood. The benefits include prevention and treatment of obesity, maintenance of normal serum lipid values, and a lower risk of developing cardiovascular diseases (48, 49). The effect of fiber is mediated, in part, by the phytosterols present in whole grains that compete with the fat for absorption in the small intestine (48). Other key factors related to alterations in lipid profile comprise sedentary behaviors and physical fitness; longitudinal studies have suggested positive associations between television viewing and screen time with cardiometabolic risk in children and adolescents (50) and inverse associations between cardiorespiratory fitness with blood lipids (51). The available data have shown that sedentary behavior and poor physical fitness are problems with a high prevalence in the Ecuadorian pediatric population (52).

Another aspect to consider is the need to perform blood lipid screening among children. Some authors suggest blood lipid screenings with this population to diminish cardiovascular risk later. However, there is no consensus on the parameters to test or the age to perform routine blood lipid screening. One approach is routine screening among children with a family history of cardiovascular diseases, while other authors support universal screening. However, the available evidence on blood lipid screening has been generated using data from high-income countries; research needs to be extended to other regions with a high prevalence of cardiovascular disease and lower access to the health system (i.e., Latin America) (53, 54).

### Abdominal adiposity

Waist circumference was positively associated with blood levels of triglycerides, SGPT, and leptin. Previous reports have found a clear positive relationship between triglycerides and waist circumference (55, 56). Excess abdominal fat causes an overflow of lipids to visceral and non-adipose tissues, a phenomenon known as lipotoxicity (57, 58). The relationship between waist circumference and SGOT among young children demonstrates an advanced inflammatory state; worryingly, the available evidence has established that the association between waist circumference-SGPT values represents a strong risk factor for pediatric non-alcoholic fatty liver disease (59, 60). Therefore, our data suggest that the children studied are already at risk of developing non-alcoholic fatty liver disease.

### Differences between urban and rural settings

Our data prove that children living in urban areas in Ecuador are at a higher risk. Dietary intake seems not to explain the differences in cardiovascular risk between urban and rural children; although the net daily energy intake was higher in the urban area, energy density estimated as E%/day did not differ between the study regions. Furthermore, the energy obtained from processed and ultra-processed foods rich in added sugar and saturated fat between urban and rural children is similar, a result in line with a previous study performed among urban and rural adolescents living in Ecuador (33); and, insufficient fiber intake is similar in both settings. Therefore, the differences might be explained by sedentary behavior, physical activity, or cardiorespiratory fitness differences. The Ecuadorian health survey performed in 2012 showed how 22% of children in the urban Andean region spent more than 2 h per day viewing television, while 15% of rural children reported more than 2 h.

Similarly, the fraction of children classified as active was lower in the urban region (21). Data regarding differences in urban and rural settings in Latin America are scarce. A recent report from Brazil shows how rural children perform better in cardiorespiratory fitness tests and have lower cardiometabolic risk (54). The lower cardiorespiratory fitness in urban settings might result from living conditions. For example, rural children and adolescents in Latin America spend more time in agriculture-related activities (54, 61). Other possible explanations involve crime concerns and distances to green spaces secondary to inadequate urban planning (54). Our data demonstrate how cardiovascular risk affects a considerable proportion of young children with a higher impact in urban settings. Formative research is needed to design effective strategies aimed at reverting the problems according to each setting; one might consider that although the risk is lower among rural children, the risk’s magnitude is important in both settings.

Adiponectin was significantly lower among children living in urban areas. Considering that urban children have higher BMI, waist circumference, and more unfavorable lipid profiles, it is reasonable that adiponectin levels are lower in this group (11). Interventions promoting physical activity among children have effectively increased adiponectin levels among obese children. Once again, this highlights the need to undertake preventive strategies to revert the children’s inflammatory state in the region (62).

This study was designed to determine cardiovascular risk factors among young children. Our findings demonstrate that young children are at high cardiovascular risk; if actions are not taken, the burden of non-communicable diseases will be substantial. The differences in risk between rural and urban areas are evident; urbanization might predispose children to a different reality and, in most cases, result in poor health habits. Adipokines, hepatic enzymes, and lipid profiles were strongly correlated with waist circumference and urbanization. Several combined factors are responsible for the current health status of the children studied. The findings highlight the need to implement preventive strategies to increase fiber intake and prevent overweight, obesity, and cardiovascular risk. Urbanization is critical when developing effective strategies tailored to the local context. This study highlights a significant problem in an understudied population (i.e., 6–8-year-old children living in Latin America) and demonstrates the need to implement comprehensive policies.

The study has some limitations. The participants were selected by convenience. Nevertheless, the results are comparable with previous reports, and this study has the strength to analyze several risk factors among young children living in urban and rural Latin America. Future studies should extend the research to larger samples. We did not measure some behaviors, such as physical activity and sedentary behavior; our data suggest that such behavior might be an influential factors.

## Data availability statement

The data that support the findings of this study are available from the corresponding authors LB-R, lybaldeon@uce.edu.ec and AO-A, angelica.ochoa@ucuenca.edu.ec, upon reasonable request. The data from the urban area used in this article formed part of a Bachelor thesis (63).

## Ethics statement

The studies involving human participants was reviewed and approved by the Comité de Ética de Investigacón en Seres Humanos de la Universidad San Francisco de Quito “CEISHUSFQ.” Written informed consent to participate in this study was provided by the participants’ legal guardian/next of kin.

## Author contributions

LB-R acquired the funding, processed the blood samples, and collected the 24-h recall data from rural areas. LB-R and AO-A designed the research. NL-V and SV-R supported the field work and collected the 24-h recall data from urban areas. SE, SV-R, NL-V, and AO-A conceived statistics analyses. AO-A, SV-R, NL-V, SE, LB-R, and CO-A were involved in interpreting results, drafting, editing the manuscript, and approving the submitted and published versions. All authors contributed to the article and approved the submitted version.

## Conflict of interest

The authors declare that the research was conducted in the absence of any commercial or financial relationships that could be construed as a potential conflict of interest.

## Publisher’s note

All claims expressed in this article are solely those of the authors and do not necessarily represent those of their affiliated organizations, or those of the publisher, the editors and the reviewers. Any product that may be evaluated in this article, or claim that may be made by its manufacturer, is not guaranteed or endorsed by the publisher.

## Footnotes

1
http://www.rstudio.com/
